# Prevalence of hearing loss in pseudohypoparathyroidism

**DOI:** 10.1186/s13023-024-03299-3

**Published:** 2024-09-12

**Authors:** Cassandre Djian, Jugurtha Berkenou, Anya Rothenbuhler, Jérémie Botton, Agnès Linglart, Jérôme Nevoux

**Affiliations:** 1 AP-HP, Department of Otolaryngology, Hôpital Lariboisière, Université Paris Cité, Paris, France; 2 AP-HP, Department of Pediatric Endocrinology, Hôpital Bicêtre Paris Saclay, Le Kremlin- Bicêtre, France; 3 AP-HP, Reference Center for Rare Disorders of the Calcium and Phosphate Metabolism, Filière OSCAR, EndoRare, BOND ERN and Platform of Expertise for Rare Diseases Paris-Saclay, Hôpital Bicêtre Paris Saclay, Le Kremlin-Bicêtre, France; 4 Faculté de Pharmacie, Université Paris Saclay, Orsay, 91400 France; 5 Université Paris Saclay, Le Kremlin-Bicêtre, France; 6 AP-HP, Department of Otolaryngology, Hôpital Bicêtre Paris Saclay, Le Kremlin-Bicêtre, France

## Abstract

**Background:**

The main clinical features of pseudohypoparathyroidism (PHP)/inactivating parathyroid hormone/parathyroid hormone-related protein signaling disorders (iPPSD), including parathyroid hormone (PTH) resistance, brachydactyly and short stature, develop during middle and late childhood. Very few studies have addressed hearing loss in PHP/iPPSD patients, and these studies have yielded widely divergent conclusions. The aim of our study was to assess hearing and determine the predictive factors of hearing loss in patients with PHP/iPPSD.

**Methods:**

Our retrospective cohort study was conducted between March 2019 and May 2020 in the Otolaryngology Department and the calcium phosphate reference center for rare diseases in Bicêtre Paris-Saclay Hospital, France. We retrospectively collected data from patients with PHP/iPPSDs (age, sex, genetic mutations, height, body mass index (BMI), PTH resistance, presence or absence of ectopic ossifications and brachydactyly). All patients underwent auditory investigations, including tonal and vocal audiometry. The primary outcome was the pure tone average (PTA). The PTA was compared with the norm according to the International Organization for Standardization. Hearing loss was defined as a PTA ≥ 20 db.

**Results:**

The median age of the patients was 15.6 years [9.5, 28.5]. Thirty-six patients were diagnosed with iPPSD2, and eight were diagnosed with iPPSD3. Twenty-six of them (59%) were female. Hearing impairment was confirmed in 17 patients (39%). The mean PTA and the mean SRT of the deaf ears were 40 ± 26 db and 31 ± 14 db. The mean difference in the PTA between the patients and the normal controls was 11.4 db (*p* = 0.00002). Short stature and the presence of ectopic ossifications were two significant predictive factors of hearing loss (*p* = 0.009 and *p* = 0.03, respectively). Sex, BMI, PTH resistance, mutation category and brachydactyly were not associated with an increased risk of hearing loss (*p* = 0.19, *p* = 0.41, *p* = 0.13, *p* = 0.50, *p* = 0.19, respectively).

**Conclusion:**

Our study confirmed the frequency of hearing loss in patients with PHP/iPPSD disease (prevalence = 39%). A diagnosis of PHP/iPPSD should trigger auditory investigations and follow-up, especially when short stature and/or ectopic ossifications are present.

## Introduction

Pseudohypoparathyroidism (PHP) belongs to a group of metabolic disorders called “inactivating parathyroid hormone (PTH)/parathyroid hormone-related protein PTHrp) signaling disorders (iPPDs)”. In the PTH/PTHrp signaling pathway, signaling is triggered when PTH binds to its receptor in target organs (kidney, bone and intestine). The activation of this pathway plays a prominent role in phosphocalcic metabolism. In PHP, resistance to PTH is defined by hypocalcemia, hyperphosphatemia and elevated PTH levels in the absence of vitamin D deficiency and renal insufficiency. Impaired activation of the PTH/PTHrp signaling pathway occurs either at the level of Gsα-protein-coupled receptors or downstream [1].

Genetic and epigenetic alterations in the GNAS locus, which encodes the α subunit of the Gsα protein, are responsible for the PHP phenotype [2]. The phenotype includes hypocalcemia and hyperphosphatemia, both due to proximal renal tubular resistance to PTH and an Albright Hereditary Osteodystrophy (AHO). Brachydactyly, ectopic ossification, early-onset obesity, and short stature are the main features of the AHO phenotype. The incidence of PHP is 0.3 to 1.1/100,000 [3]. PHPs are classified as PHP1a, 1b, 1c or 2 according to their clinical and biological features. The classification of iPPSDs based on genetic mutations has recently been proposed [4].

The association between PHP and hearing loss has not been confirmed, and the mechanism of hearing loss is poorly known. Conductive hearing loss may result from the craniofacial malformations associated with PHP, which, by causing serous otitis media, frequently requires the use of tympanostomy tubes [5]. Sensorineural hearing loss could be explained by the fact that the cochlear G proteins and the adenylate cyclase of the inner ear share properties with the PTH signaling pathway [6]. Very few studies have been dedicated to the subject of hearing loss in PHP, and these studies have yielded largely divergent conclusions, presumably because of the small size of the cohorts used [6–8]. The objective of our study was to demonstrate that sensorineural hearing loss is a common characteristic of pseudohypoparathyroidism and to discuss the underlying pathophysiological mechanisms involved. Our study has practical implications for the follow-up of patients with PHP.

## Patients and methods

### Patients and characteristics of the study

A retrospective observational study was conducted between March 2019 and May 2020 in the otolaryngology, pediatric and adult endocrinology departments of a tertiary referral center in Paris, France. Children and adults affected by either iPPSD2 or iPPSD3 who were followed in the pediatric and adult departments of endocrinology were consecutively included. The diagnostic criteria for iPPSD2 and iPPSD3 included an AHO phenotype associated with resistance to PTH and a loss-of-function mutation in either the coding region of the GNAS gene (iPPSD2) or its promoter (iPPSD3) [9]. Patients with a strong suspicion of iPPSD2 or 3 (AHO phenotype associated with PTH resistance with exclusion of differential diagnoses) were included. Patients who could not speak French were excluded from the study. The primary outcome was the pure-tone average (PTA), which was calculated as the average of the hearing thresholds at frequencies of 500, 1000, 2000 and 4000 Hz. The secondary outcomes were the speech recognition threshold (SRT), the presence or absence of acoustic-otoemissions (AOE) and the wave latency of the acoustic brainstem response (ABR). This study was conducted in our department in accordance with the Declaration of Helsinki, Good Clinical Practice guidelines, and local laws and regulations. Patients and/or their parents/legal guardians were informed verbally of the objectives and procedures of the study, and their verbal consent was obtained. They have the right to refuse to participate or to withdraw at any time by writing to http://recherche.aphp.fr/eds/droit-opposition. The study complied with the CNIL recommendations for the collection and use of patient data, and the registration number in the general registry DPO (Data Protection Officer) APHP Paris-Saclay is 20,220,824,133,612.

### Data collection

General, audiological, and iPPSD-related data were collected. The iPPSD-related data included sex, height, body mass index (BMI), ectopic ossifications, PTH resistance, mutation category and brachydactyly. Audiological data included neonatal hearing screening, history of otitis media or tympanostomy tubes or otologic surgical procedures, walking age, and history of balance disorders. iPPSD-related data included genetic mutation, height, BMI, PTH resistance, the presence or absence of ectopic ossifications and brachydactyly. The height and BMI are expressed as the standard deviation from the norm [10]. All patients with confirmed iPPSD were referred to an otologist for an otologic examination as well as audiometric and electrophysiological tests.

### Auditory investigations

Audiometry: Audiometry was performed in a soundproof booth by an otologist specializing in audiology. Air and bone conduction hearing thresholds were determined between 125 and 8000 Hz per octave frequency (125, 250, 500, 1000, 2000, 4000 and 8000 Hz). The contralateral ear was masked if necessary. Adult audiograms were obtained using a Madsen Astera audiometer (Otometrics Natus Medical Denmark). Pediatric audiograms were obtained by a pediatric audiologist using a Clinical Audiometer AC30 (Interacoustics, Denmark). Speech audiometry was measured with the same device to determine intelligibility thresholds. Fournier’s dissyllabic lists for adults and Boorsma’s lists for children were used to determine intelligibility thresholds. Sensorineural hearing loss was defined as an equal decrease in average air and bone hearing thresholds below or equal to 20 db. Conductive hearing loss was defined as a decrease in the average air threshold below or equal to 20 dB with a Rinne of 5 dB or more. Hearing loss was classified according to the pure tone average (PTA) as mild (20–40 dB), moderate (41–70 dB), severe (71–90 dB) or profound (> 90 dB). Hearing thresholds were also analyzed by frequency. In the case of no response, a threshold of 120 dB was established for statistical analysis.

Acoustic otoemissions: These events were performed bilaterally in patients at rest in a quiet room at frequencies of 1000, 1500, 2000, 3000 and 4000 Hz. Otoemissions were positive when they were detectable at 3 out of 5 frequencies. They were measured with a NeuroAudio device (Neurosoft, Russia).

Auditory brainstem responses: ABRs were measured bilaterally in a patient at rest in a quiet room to avoid noise interference using a NeuroAudio device (Neurosoft, Russia). Melatonin was premedicated before the age of four to allow for a better-quality recording. After checking the impedances, sound stimulation by clicks at different intensities (70, 50, 30 and 20 dB) allowed the collection of a summation potential of 2000 acquisitions. I-wave latencies and I-V latencies were collected and compared to the standard norm [11].

### Statistical analysis

Quantitative variables describing patient characteristics, including age (years), height (z score), and BMI (z score), are expressed as medians with interquartile ranges [IQRs] (ranges). Qualitative data are described as numbers and percentages (%). Univariate analysis was performed to estimate potential predictive factors of hearing loss in our cohort. We used Student’s t test or the Mann‒Whitney test or the chi‒squared test to compare quantitative and qualitative variables, respectively, when analyzing the differences between the iPPSD/PHP group with hearing loss and the iPPSD/PHP group without hearing loss. The PTA was compared to the normal value (median according to the ISO standard) using Student’s t test. Clinical predictive factors of hearing loss were assessed using a generalized estimating equation model. Variables associated with an outcome with a p value less than 0.20 were added to the multivariable analysis, except age, which was highly correlated, leading to collinearity issues and PTH resistance highly related to the other characteristics of the disease. Sex was also maintained as a major variable in the multivariable model. Statistical analyses were performed using R software version 4.0.3. The significance threshold (p value) was set at 0.05.

## Results

### Patients

None of the patients who were approached refused to be included in the study. We collected data from 44 patients affected with iPPSD/PHP. All patients were followed on a regular basis at the endocrinology and diabetes department for children or at the endocrinology adult department of Bicêtre Paris Saclay Hospital (Le Kremlin Bicêtre - Paris, France). The audiological assessments were carried out at the otolaryngology department of the same hospital. The demographic and clinical features of the patients are reported in Table 1. A total of 44 patients were included, 26 of whom were children and 18 of whom were adults. The median age was 15.6 [9.5, 28.5] years. There were 26 females and 18 males. Thirty-six patients (82%) were diagnosed with iPPSD2, and eight were diagnosed with iPPSD3 (18%).

**Table 1:** Demographical and clinical features of patients by iPPSD subtypes

Subtype of iPPSD	iPPSD2	iPPSD3	Total
Patients count (%)	36 (82%)	8 (18%)	44 (100%)
Sex : F(%)	22 (61%)	4 (50%)	26 (59%)
Median age [Q1, Q3] years (range)	**14.5** [9.3, 25.9] (3.5, 52.9] NA = 0	**28.1** [19.0, 36.8] (11.8, 56.3) NA = 0	**15.6** [9.5, 28.5] (3.5, 56.3) NA = 0
Median height [Q1, Q3] z-score (range)	**-0.9** [-2.4, 0.9] (-4.1, 2.3) NA = 1	**0.5** [-1.1, 1.0] (-1.5, 2.2) NA = 1	**-0.8** [-1.6, 0.9] (-4.1, 2.3) NA = 2
Median BMI [Q1, Q3] z-score (range)	**2.0** [0.6, 2.9] (-1.6, 5.2) NA = 3	**2.0** [0.6, 2.5] (-0.7, 3.6) NA = 1	**2.0** [0.5, 2.9] (-1.6, 5.2) NA = 4
Resistance to PTH	28 (78%) NA = 2	7 (88%) NA = 1	35 (80%) NA = 3
GNAS mutation	34 (94%) NA = 2	0 (0%) NA = 3	34 (77%)
Ectopic ossification (Count/%)	26 (72%) NA = 2	0 (0%) NA = 2	26 (59%) NA = 4
Brachydactily	27 (75%) NA = 2	1 (12%) NA = 2	28 (64%) NA = 4

### History and otological symptoms

No patient reported a history of balance disorders. Walking age was delayed in one patient, and one patient reported negative hearing screening at birth. Seven patients had a history of tympanostomy tube insertion. Clinical examination was normal for all patients. One patient had a history of middle ear surgical exploration for malleus head fixation. No patient reported a history of otological risk factors.

### Audiologic assessment

**Fig. 1:**
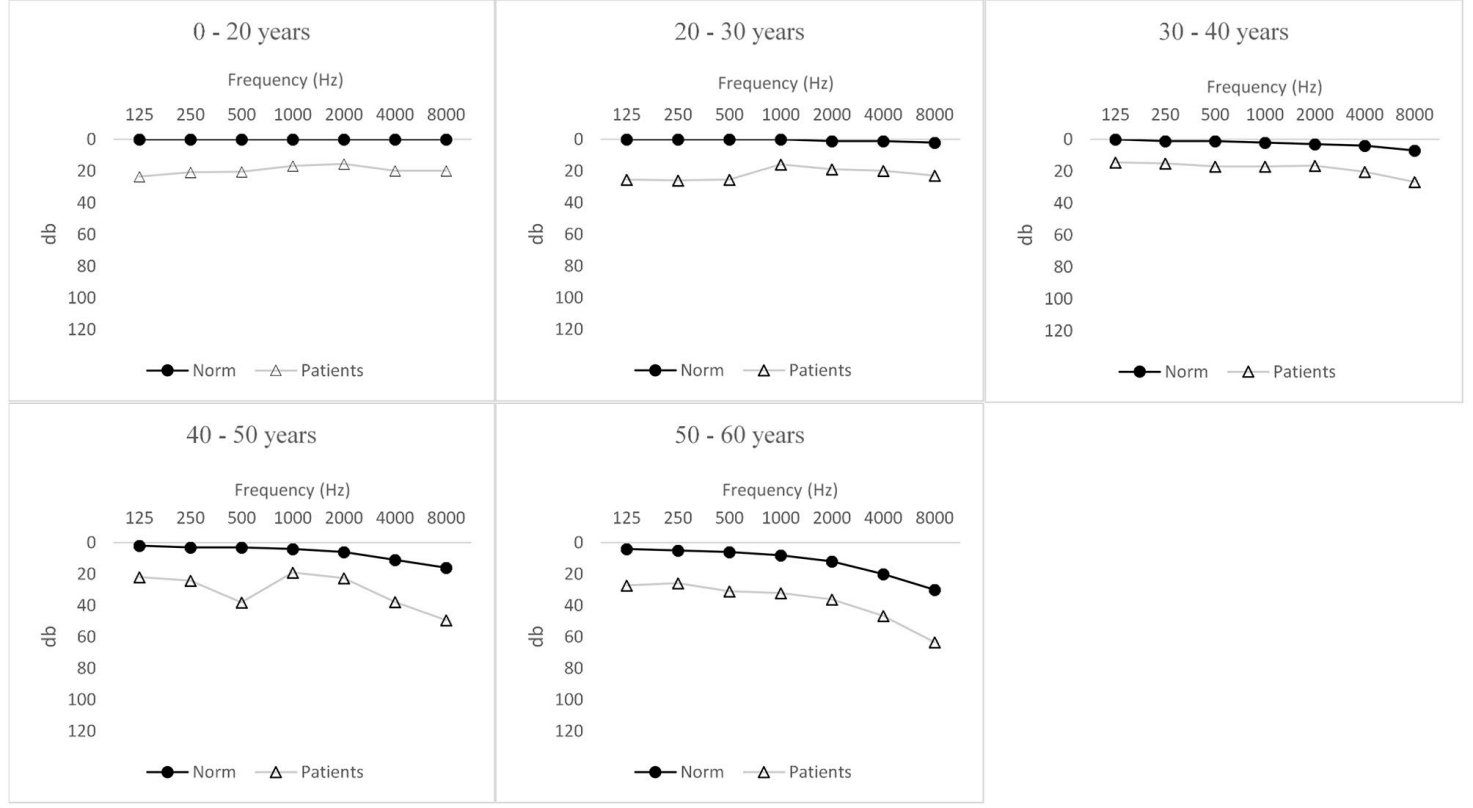
Mean hearing thresholds by age and frequency of patients with pseudohypoparathyroidism and standards from the International Organization for Standardisation

Audiometry: The audiometric results are reported in Table 2. Eighty-eight ears were used for audiogram, AOE and ABR analyses. Hearing impairment, as defined in the Methods section, was confirmed in 17 patients (39%) and 26 ears (30%). Hearing loss was unilateral in eight patients and bilateral in nine patients. The mean PTA and the mean SRT of the deaf ears were 40 ± 26 db and 31 ± 14 db, respectively. Hearing loss was bilateral in nine patients and unilateral in eight patients. Among these 26 ears, hearing loss was mild in 15 ears (17%), moderate in nine ears (10%) and profound in two ears. Hearing thresholds by frequency and age group are reported in Fig. 1. There were no vocal distortions. The mean difference in the PTA between the patients and the normal controls was 11,4 db (*p* = 0.00002).

**Table 2:** Patients’ characteristics

Patient	Age	Gender	iPPSD	PTA		SRT		AOE		I – V wave latency (ABR)	
				Right	Left	Right	Left	Right	Left	Right	Left
**1**	3.5	M	2	21	21	MD	MD	1	1	5	3.88
**2**	5.5	M	2	120	20	MD	10	0	1	Abs	Abs
**3**	6.3	M	2	14	13	12	10	1	1	3.81	3.88
**4**	6.8	F	2	36	41	34	29	1	1	3.67	3.57
**5**	7.3	F	2	20	50	20	MD	1	1	3.63	4.08
**6**	7.8	F	2	11	21	31	31	1	1	Abs	Abs
**7**	9.1	M	2	5	6	5	7	1	1	4.18	4.2
**8**	9.2	M	2	15	9	12	5	1	1	3.65	3.8
**9**	9.3	F	2	30	30	20	21	1	1	Abs	Abs
**10**	9.3	F	2	10	13	0	2	MD	MD	MD	MD
**11**	9.3	F	2	11	10	3	1	1	1	4.28	4.18
**12**	9.6	F	2	10	8	-8	-4	1	1	MD	MD
**13**	9.6	F	2	11	9	10	12	1	1	3.75	3.94
**14**	10	M	2	45	11	40	10	MD	MD	MD	MD
**14**	10.5	F	2	13	11	2	4	1	1	3.85	3.57
**15**	11.8	F	3	34	9	23	6	MD	MD	MD	MD
**16**	11.9	M	2	13	11	21	26	1	1	4.28	4.19
**18**	12	M	2	4	11	20	21	1	1	4.19	4.19
**19**	12.8	F	2	13	18	9	9	1	1	3.6	3.67
**20**	13.9	M	2	18	16	25	26	1	1	4.2	3.85
**21**	15	F	2	30	10	22	2	MD	MD	MD	MD
**21**	15.5	F	2	15	10	4	-2	1	1	4.05	4.2
**23**	15.7	M	2	6	15	5	18	MD	MD	MD	MD
**24**	16	M	3	9	9	0	0	MD	MD	MD	MD
**25**	17.3	F	2	6	6	19	15	1	1	3.97	3.77
**26**	17.8	M	3	6	5	13	15	1	1	4.2	4.3
**27**	18.5	M	3	15	10	5	5	1	1	4.1	4.15
**28**	21.1	M	2	15	8	4	20	1	1	4.13	4.88
**29**	23.3	F	2	6	9	21	17	1	1	4	3.9
**30**	25.2	F	2	60	66	48	50	0	0	3.98	3.93
**31**	25.2	M	2	21	13	23	25	1	1	3.91	4.38
**32**	28	F	2	1	15	20	23	1	1	4.07	4.17
**33**	28.2	F	2	24	24	32	27	1	1	3.93	4.12
**34**	29.4	F	2	15	11	12	8	1	1	4.03	3.85
**35**	31.9	F	2	13	6	14	12	1	1	3.28	4.03
**36**	32.9	F	2	15	43	12	20	0	0	4.1	3.78
**37**	34.5	M	3	3	4	5	5	1	1	4.03	3.93
**38**	35.3	F	2	23	26	35	35	0	0	3.57	Abs
**39**	38.8	F	2	11	10	1	1	1	1	3.63	3.88
**40**	41.3	F	3	16	14	22	24	1	1	3.78	Abs
**41**	48.7	F	2	28	120	25	MD	0	0	4.76	Abs
**42**	52.6	F	2	19	23	8	6	1	1	3.97	3.72
**43**	52.9	F	3	44	43	60	55	1	1	Abs	Abs
**44**	56,3	M	2	5	18	2	6	0	0	4.06	4.47

PTA pure-tone average. SRT speech recognition threshold. AOE acoustic otoemissions. ABR auditory brainstem response.

Abs absent wave I. MD missing data

Acoustic otoemissions were carried out on 76 ears. Acoustic otoemissions were present in 65 ears and absent in 11 ears. Otoemissions were measured in 23 of the 26 deaf ears; otoemissions were present in 14 ears and absent in 9 ears.

Auditory brainstem response: Data were missing for 16 ears, and wave I was absent in 9 ears. The mean I-wave latency and mean I-V latency were calculated for 63 ears. Across all ears, the mean I-wave latency was 1,7 ± 0.28 ms, and the mean I-V latency was 3.87 ± 0.14 ms. Among the deaf ears, the mean I-wave latency was 1,72 ± 0,29 ms, and the mean I-V latency was 3,99 ± 0,39 ms.

### Predictive factors of hearing loss

Short stature and the presence of ectopic ossifications were two significant predictive factors of hearing loss (*p* = 0.01 and *p* = 0.03, respectively). Sex, BMI, PTH resistance, mutation category and brachydactyly were not associated with an increased risk of hearing loss (*p* = 0.19, *p* = 0.41, *p* = 0.13, *p* = 0.50, *p* = 0.19, respectively) (Table 3).

**Table 3:** Univariable and multivariable analysis: predictive factor of hearing loss (HL) in our cohort of 44 patients diagnosed with iPPSD. Correlation between hearing loss and main clinical features of the disease

Variable	Group 1: Hearing loss = yes (*N* = 17 )	Group 2: Hearing loss = no (*N* = 27 )	*p* value (univariable analysis)	*p* value (multivariable analysis)
Sex: F (%)	12 (70%)	14 (52%)	0.21	0.19
Median age [Q1, Q3] years	15.5 [9.2, 28.2] NA = 0	16.0 [9.8, 27.3] NA = 0	0.50	**0.04**
Median height [Q1, Q3] z-score	-1.46 [-3.4, -0.8] NA = 1	0.0 [-1.2, 1.0] NA = 1	**0.005**	**0.009**
Median BMI [Q1, Q3] z-score	1.1 [-0.5, 2.8] NA = 1	2.3 [1.3, 2.9] NA = 3	0.2	0.41
PTH resistance	13 (76%) NA = 1	22 (81%) NA = 2	0.6	0.13
GNAS mutation	16 (94%) NA = 0	18 (67%) NA = 5	0.3	0.50
Ectopic ossifications	13 (76%) NA = 2	13 (48%) NA = 2	**0.02**	**0.03**
Brachydactily	13 (76%) NA = 2	15 (56%) NA = 2	0.07	0.19

## Discussion

To our knowledge, this is the first study aimed at investigating hearing in patients with pseudohypoparathyroidism, a rare genetic disorder affecting phosphocalcic metabolism. In our cohort of 44 patients, the prevalence of hearing impairment was 39%. The mean hearing threshold was 11.4 dB lower than the ISO standard. The fact that only one patient presented with conductive hearing loss indicated that PHP was not a risk factor for middle ear or Eustachian tube dysfunction. In all but one patient, perceptive hearing loss could be a sign of endocochlear, synaptic or retrocochlear damage. ABR measurements revealed that I-V latencies were conserved, whereas I-wave latencies were slightly increased [11]. These results associated with correlated PTA and SRT were in favor of either endocochlear or synaptic damage with normal function of the cochlear nerve. Hearing loss was more common unilaterally than bilaterally and ranged from mild to profound; all frequencies were similarly affected. We noticed that the difference between the hearing thresholds of the patients and those of the subjects who met the ISO standard was stable regardless of age. This observation suggested either nonprogressive hearing loss or hearing stabilization by long-term hormonal and calcic replacement therapy. However, the influence of calcic and hormonal therapy was not analyzed in our study.

Since all patients showed normal hearing at birth, we suggest that hearing impairment developed during the first few years of life along with the AHO phenotype. No correlation of hearing function with sex, weight, PTH resistance, mutation category or brachydactyly was found. The presence of ectopic ossifications and short stature were the only two variables associated with an increased risk of hearing loss. Within the AHO phenotype, clinical features are associated with different pathophysiological mechanisms. Short stature, brachydactyly and ectopic ossification are due to reduced Gs alpha protein activity, resulting in altered chondrocyte and osteocyte activity in the tissues. These clinical features appear to be independent of calcium or phosphate levels [1]. PTH resistance leads to a dysfunction of the phosphocalcic metabolism and is responsible for hypocalcemia and hyperphosphatemia. The pathophysiology of hearing loss remains elusive as hearing loss appears to be associated with ectopic ossification and short stature, but not with PTH resistance and brachydactyly. Therefore, it is not clear whether hearing loss is due to impaired function of the Gs alpha protein, dysregulation of phosphocalcic metabolism, or some other cause. It is worth noting that the small size of the cohort and the limited power of the results may be an obstacle to the analysis of the results. The lack of significance of the gender factor could similarly be explained by the small size of the cohort, with 59% of the patients being female and 41% male.

The main limitation of our study was the absence of a sex- and age-matched control group. Assembling a control group poses an ethical problem, particularly for the execution of auditory evoked potentials, which may require premedication in infants. The existence of a control group would have been particularly useful for the pediatric population, for which audiometric standards do not exist before 20 years of age. To avoid overestimating hearing loss in children, the normal reference threshold was homogenized with the hearing thresholds of 20-year-old adults according to the ISO standard. Another limitation of our study was the number of patients lost during the endocrinological follow-up, as these patients had to be removed from the study. The greater likelihood of patients quitting medical follow-up when their hearing was unaffected might have led us to overestimate the prevalence of hearing loss.

We conclude that audiological follow-up should be routinely carried out in patients with pseudohypoparathyroidism regardless of the severity of the disease. At the time of diagnosis, patients should all be tested by tone and speech audiometry, and an annual audiometric follow-up should be integrated into the general follow-up of patients with hearing loss, particularly in cases of short stature and ectopic ossifications. In the case of acute or rapidly worsening symptoms, patients should be monitored at shorter intervals. In further studies, imaging of the inner ear should help clarify the pathophysiological mechanisms of hearing loss in PHP. Computed tomography will rule out calcifications or congenital malformations of the inner ear. Magnetic resonance imaging with delayed FLAIR sequences that capture the precise state of the endolymphatic and perilymphatic spaces will aid in understanding the mechanisms of endocochlear damage [12]. Although we did not detect clinical alterations in vestibular function, a full vestibular work-up could help clarify the topography of lesions of the inner ear.

## Conclusion

The prevalence of hearing loss in patients with PHP was found to be 39%. The mean difference in the PTA between the patients and the normal controls was 11.4 db. The most frequent presentation was a sensorineural nonevolutive unilateral or bilateral hearing loss affecting all frequencies. Endocochlear damage was the most likely pathophysiological mechanism. CT and MRI of the inner ear and a complete vestibular assessment should help clarify the pathophysiological mechanisms of the hearing impairment associated with PHP. An auditory assessment at the time of diagnosis as well as an annual follow-up need to be added to the global management of patients with PHP.

## Data Availability

The authors confirm that the data supporting the findings of this study are available within the article.

## Abbreviations

ABR: Auditory brainstem response
AHO: Albright hereditary osteodystrophy
AOE: Acoustic otoemissions
BMI: Body mass index
iPPSD: inactivating PTH/PTHrp signaling disorder
PHP: Pseudohypoparathyroidism
PTA: Pure-tone average
PTH: Parathyroid hormone
SRT: Speech recognition threshold
